# Novel Missense Mutation A789V in *IQSEC2* Underlies X-Linked Intellectual Disability in the MRX78 Family

**DOI:** 10.3389/fnmol.2015.00085

**Published:** 2016-01-11

**Authors:** Vera M. Kalscheuer, Victoria M. James, Miranda L. Himelright, Philip Long, Renske Oegema, Corinna Jensen, Melanie Bienek, Hao Hu, Stefan A. Haas, Maya Topf, A. Jeannette M. Hoogeboom, Kirsten Harvey, Randall Walikonis, Robert J. Harvey

**Affiliations:** ^1^Department of Human Molecular Genetics, Max Planck Institute for Molecular Genetics Berlin, Germany; ^2^Research Group Development and Disease, Max Planck Institute for Molecular Genetics Berlin, Germany; ^3^Department of Pharmacology, UCL School of Pharmacy London, UK; ^4^Department of Physiology and Neurobiology, University of Connecticut Storrs, CT, USA; ^5^Department of Clinical Genetics, Erasmus MC University Medical Center Rotterdam Rotterdam, Netherlands; ^6^Department of Computational Molecular Biology, Max Planck Institute for Molecular Genetics Berlin, Germany; ^7^Department of Biological Sciences, Institute for Structural and Molecular Biology, Birkbeck College London, UK

**Keywords:** ArfGEF, BRAG1, IQ-ArfGEF, IQSEC2, MRX78, XLID

## Abstract

Disease gene discovery in neurodevelopmental disorders, including X-linked intellectual disability (XLID) has recently been accelerated by next-generation DNA sequencing approaches. To date, more than 100 human X chromosome genes involved in neuronal signaling pathways and networks implicated in cognitive function have been identified. Despite these advances, the mutations underlying disease in a large number of XLID families remained unresolved. We report the resolution of MRX78, a large family with six affected males and seven affected females, showing X-linked inheritance. Although a previous linkage study had mapped the locus to the short arm of chromosome X (Xp11.4-p11.23), this region contained too many candidate genes to be analyzed using conventional approaches. However, our X-chromosome exome resequencing, bioinformatics analysis and inheritance testing revealed a missense mutation (c.C2366T, p.A789V) in *IQSEC2*, encoding a neuronal GDP-GTP exchange factor for Arf family GTPases (ArfGEF) previously implicated in XLID. Molecular modeling of IQSEC2 revealed that the A789V substitution results in the insertion of a larger side-chain into a hydrophobic pocket in the catalytic Sec7 domain of IQSEC2. The A789V change is predicted to result in numerous clashes with adjacent amino acids and disruption of local folding of the Sec7 domain. Consistent with this finding, functional assays revealed that recombinant IQSEC2^A789V^ was not able to catalyze GDP-GTP exchange on Arf6 as efficiently as wild-type IQSEC2. Taken together, these results strongly suggest that the A789V mutation in *IQSEC2* is the underlying cause of XLID in the MRX78 family.

## Introduction

Intellectual disability (ID) is a developmental brain disorder characterized by impaired intellectual and adaptive functions, and can be defined by an IQ below 70 and limitations in intellectual functioning and adaptive behaviors. As a result of the excess in males affected by ID, and the identification of families where ID shows clear X-linked segregation, significant attention has focused on the genetics of X-linked intellectual disability (XLID)—a common, clinically and genetically complex disorder often arising from mutations in one of >100 genes on the X chromosome. XLID may be associated with other clinical, morphological, or behavioral symptoms (syndromic XLID) or appear without other associated defects (non-syndromic XLID). However, despite extensive genetic studies using conventional linkage/candidate gene analysis or analysis of copy number variants (CNVs), for a significant number of families, the underlying cause of XLID has remained unclear. Fortunately, the genetics of XLID has recently been accelerated by next-generation DNA sequencing and novel bioinformatics approaches for variant filtering (de Ligt et al., 2012; Rauch et al., 2012; Redin et al., 2014; Hu et al., 2015; Tzschach et al., 2015). However, analysis of large exome data sets from the general population has also revealed a number of “XLID genes” where truncating variants or previously published “mutations” are observed at a relatively high frequency in normal controls, calling into question whether they are indeed causal. For example, recent studies questioned the implication of *AGTR2*, *MAGT1, ZNF674, SRPX2, ATP6AP2, ARHGEF6, NXF5, ZCCHC12*, *ZNF41*, *ZNF81* and *RAB40AL* in XLID (Piton et al., 2013; Ołdak et al., 2014). This highlights the vital importance of structure-function analyses for validating potentially disease-causing variants.

In this study, we have combined next-generation sequencing, variant filtering and structure-function assays to resolve the cause of XLID in a large family known as MRX78 (de Vries et al., 2002). A previous investigation of the MRX78 family revealed linkage to the short arm of chromosome X (Xp11.4-p11.23; de Vries et al., 2002). We present compelling evidence that the likely cause of XLID in the extended MRX78 pedigree with six affected males and seven affected females in four generations is a missense mutation in the known XLID gene *IQSEC2* (MIM*300522; Shoubridge et al., 2010) encoding a neuronal ArfGEF (known as IQSEC2, BRAG1 and IQ-ArfGEF) involved in cytoskeletal organization, dendritic spine morphology and excitatory synaptic organization, along with a review of previously published *IQSEC2*-related XLID patients.

## Materials and Methods

### Genetic Analysis

Written informed consent was obtained from the legal guardians of patients III-5 and III-8 regarding next-generation sequencing. This study was declared exempt from approval by the medical ethics committee of the Erasmus MC University Medical Center (decision MEC-2012-387). Confirmation of the mutation in the additional family members was performed on DNA previously obtained and used for diagnostic purposes and was carried out according to Erasmus MC University Medical Center regulations for secondary use of tissue after diagnostic procedures. In addition, patients or legal guardians were informed and gave verbal consent to use remaining samples for scientific research. X-chromosome exome resequencing and bioinformatics analysis was performed as recently described (Hu et al., 2014, 2015). DNA from the affected male individual III-8 was used for constructing the sequencing library using the Illumina Genomic DNA Single End Sample Prep kit (Illumina, San Diego, CA, USA). Enrichment of the X-chromosomal exome was then performed using the Agilent SureSelect Human X Chromosome Kit (Agilent, Santa Clara, CA, USA), which contains 47,657 RNA baits for 7591 exons of 745 genes of the human X chromosome. Single-end deep sequencing was performed on the Illumina Genome Analyzer GAIIx (Illumina, San Diego, CA, USA). Reads were subsequently mapped to the human reference genome (hg18 without random fragments) with RazerS (Weese et al., 2009) tolerating a sequence difference up to 5 bp per read. We applied the split mapping tool SplazerS (version1.0; Emde et al., 2012) in order to detect short insertions (<30 bp) and larger deletions (<50 kb) from unmapped and indel-containing reads. In addition, large insertions/deletions were predicted using ExomeCopy (Love et al., 2011) by analysing changes in depth of coverage along the targeted regions. Single-nucleotide polymorphisms (SNPs) and short indels (<5 bp) were called with snpStore. In parallel, we used the Medical Resequencing Analysis Pipeline (MERAP; Hu et al., 2014). Here, the mapping was performed using SOAP2 allowing at most two mismatches, and requiring at least four reads for single-nucleotide variant (SNV) and indel detection. For both approaches all sequence variants were screened against public databases [dbSNP138, 1000 Genomes project, Exome Variant Server (ESP6500)], and the in-house database of the Max Planck Institute, Berlin for annotating likely non-pathogenic and previously reported neutral variants. In addition, the OMIM catalog and the Human Gene Mutation Database (HGMD) were used as a filter to identify all previously described mutations. PCR primers for mutation confirmation and segregation analysis were IQSEC2-D221F 5′-cctcttgctgtccttttcca-3′ and IQSEC2-D221R 5′-tgggcccaaaattagttcaa-3′.

### Building a Homology Model of Human IQSEC2

Structural templates for homology modeling of IQSEC2 were determined using HHPred^1^ (Söding et al., 2005). Homology modeling of the Sec7 and PH domains of IQSEC2 relied on three template crystal structures. The human BIG1 SEC7 domain (PDB ID: 3LTL, sequence identity 42%) was used to model the SEC7 domain, human GEP100 IQ motif (PDB ID: 3QWM, sequence identity 70%) was used to model the PH domain and finally, the mouse Grp1 Arf GTPase exchange factor (PDB ID: 2R0D; DiNitto et al., 2007) was used to model parts of both the Sec7 and PH domains and to guide the orientation of the domains relative to each other (overall sequence identity of 38%). A model of the 24 amino acid linker between the SEC7 and PH domains (residues 942–966) was calculated using I-TASSER^2^ (Zhang, 2008; Roy et al., 2010, 2012), which predicts proteins structure from sequence using multiple threading alignments via LOMETS (Wu and Zhang, 2007) and iterative assembly simulations based on structural templates or by *ab initio* modeling. For this linker, I-TASSER built a model with a straight α-helix, based on structural coordinates from various homologous structures in PDB (PDB IDs: 3CC2G, 2KEGA, 1K73I, 1S72G, 1M90I, 1QVFG) with sequence similarities of 21–28% [confidence score (C-score) of −0.9]. This was consistent with secondary structure prediction methods; PSIPRED^3^ (Jones, 1999) and RaptorX^4^ (Källberg et al., 2012), which predicted residues 948–960 to form a helix with a high confidence (>80%). Single pairwise, sequence-structure alignments were calculated using HHPred, then the alignments were combined manually to generate one multiple alignment of the entire IQSEC2 sequence with the four template structure sequences (three PDB structures and one model of the linker). Based on this alignment, 30 models were generated using MODELLER-9.10 and assessed with the Discrete Optimized Protein Energy (DOPE) statistical potential score (Shen and Sali, 2006). The model with the lowest DOPE score was selected as representative. The p.A789V mutation was modeled with the *swapaa* command in Chimera (Pettersen et al., 2004) using the Dunbrack backbone-dependent rotamer library (Dunbrack, 2002) and taking into account the lowest clash score, highest number of H-bonds and highest rotamer probability.

### Site-Directed Mutagenesis and Expression Constructs

The missense mutation c.C2366T, p.A789V was introduced into full-length human pCAGGS-IQSEC2 using the QuikChange site-directed mutagenesis kit (Agilent) and the primers hIQSEC2-A789V1 5′-accggtgggagtggttcacttcatcctgg-3′ and hIQSEC2-A789V2 5′-ccaggatgaagtgaaccactcccaccggt-3′. Expression constructs were verified by Sanger DNA sequencing of the entire coding region.

### Arf Activation Assay

Golgi-localized, ear-containing ARF-binding protein 3 (GGA3) pull-down of activated ARF GTPases was performed as previously described in Shoubridge et al. (2010). Plasmids encoding HA-ARF6 in pXS and FLAG-tagged wild-type IQSEC2, IQSEC2^A789V^ or IQSEC2^E849K^ in pCAGGS were co-transfected into HEK293 cells with Lipofectamine 2000 (Invitrogen, Carlsbad, CA, USA). Cells were harvested in lysis buffer (50 mM Tris-HCl, pH 7.5, 100 mM NaCl, 2 mM MgCl_2_, 0.1% SDS, 0.5% sodium deoxycholate, 1% Triton X-100, 10% glycerol, and HALT protease inhibitor cocktail (Pierce, Rockford, IL, USA), and lysates were incubated with GST:GGA3 coupled to glutathione beads. Beads were washed in lysis buffer without protease inhibitors and boiled in SDS-PAGE buffer. Samples were run on SDS-PAGE gels and transferred to PVDF membranes. The membranes were probed with primary antibodies against HA (Covance, Princeton, NJ, USA) or FLAG (Sigma, St. Louis, MO, USA) and IRDye secondary antibodies and visualized and quantified with the use of a LiCor Odyssey Infrared Imaging System. The fluorescence intensity of each of the bands was quantified with Image Studio Lite. ARF6-GTP bands were normalized to total ARF6 expression for each of the conditions, and then the ratio of normalized ARF6-GTP to normalized ARF6 was calculated for wild-type IQSEC2, IQSEC2^A789V^ and IQSEC2^E849K^. The GGA pulldown assay was assessed by one-way ANOVA followed by Tukey’s Multiple Comparison Test for *post hoc* analysis.

## Results

### Clinical Re-assessment of Family MRX78

**Figure 1:**
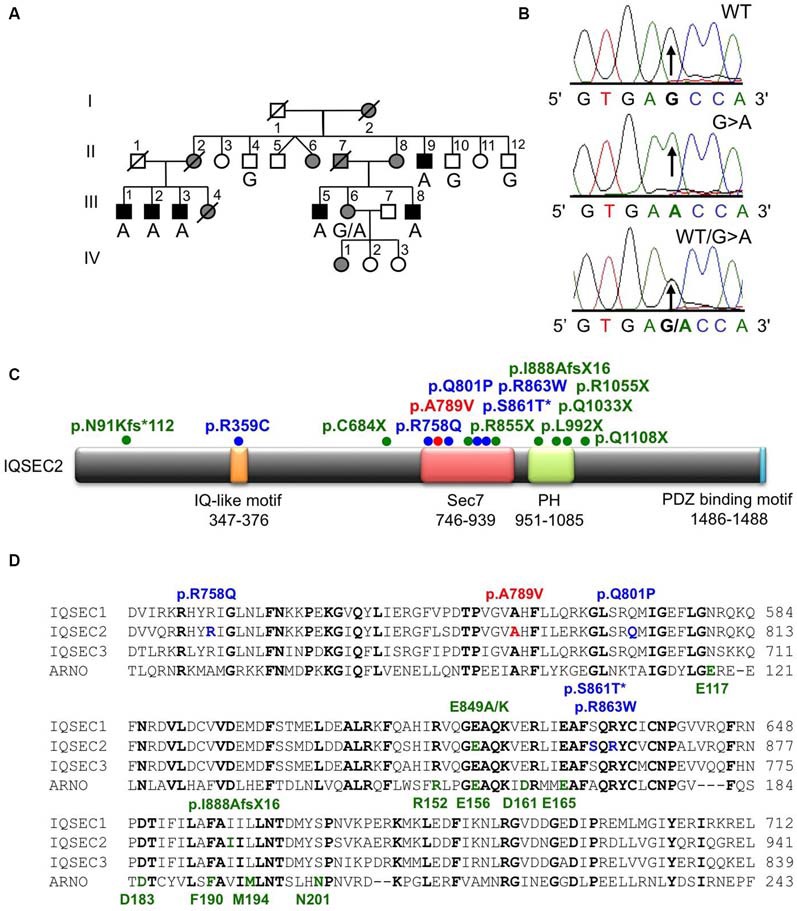
**Identification of an A789V mutation in *IQSEC2* in family MRX78. (A)** Pedigree of the MRX78 family, which has been updated. Open symbols represent normal individuals, filled gray square represents a male with learning disabilities, filled black squares represent more severely affected males, filled gray circles represent affected females. Individual generations are numbered with Roman numerals on the left of each pedigree. Individuals tested for the nucleotide substitution in each family are indicated either A (mutant allele) or G (normal allele). **(B)** DNA sequence electropherograms for the chrX:53277996 G>A mutation reported in this study. **(C)** Schematic of the human IQSEC2 protein with a regulatory IQ-like motif, a catalytic Sec7 domain, a pleckstrin homology (PH) domain and a PDZ binding motif. The relative locations of all currently known mutations in IQSEC2 are shown (see also Table 2), *indicates that p.S861T is predicted to abolish a splice site. **(D)** Sequence alignments of IQSEC1, IQSEC2 and IQSEC3 showing the location of missense mutations in the Sec7 domain. Note that IQSEC2 Sec7 domain mutations do not affect predicted GTPase binding residues (bold green type below alignments) as defined in the structure of the related Sec7 domain in the ArfGEF ARNO. Bold type indicates amino acids that are identical in all four sequences.

The revised pedigree of the MRX78 family is depicted in Figure 1A and a summary with clinical findings is presented in Table 1. Individual I-2 had mild ID and was illiterate. She had a stroke at age 71 and developed dementia thereafter. She passed away at age 80. She gave birth to 15 children; two were stillborn. Two sons and a daughter died in childhood, they were said to be “handicapped”, one son had spina bifida. All children were placed in foster homes. She had two brothers with normal cognition and three sisters. II-2 had mild ID with difficulty reading and writing. II-6 has ID. II-8 has learning difficulties and is unable to read or write. Her chromosome studies were normal and urine analysis was negative for MAO-A deficiency. Her husband (II-7) also had learning difficulties/ID. II-9 has severe ID, intractable epilepsy and does not speak, or interact socially. He does like physical contact (e.g., holding hands) and responds to his name. He developed walking difficulties in his fifties, with unstable gait (“as a drunk person”) and is now wheelchair bound. There is no tremor and his vision and hearing are good. III-1 and III-2: These brothers both have severe ID. They do not seem to recognize a familiar person and have no speech. They can be physically aggressive towards caretakers, and both are on levomepromazine therapy. Vision and hearing are normal. Urine metabolic investigations were normal, and there was no MAO-A deficiency. Subject III-1 has had one seizure as a teenager. III-3 has moderate ID and no seizures. His height is 176.5 cm, occipital frontal circumference (OFC) 55 cm, and he has no dysmorphic features. Urine metabolic investigations were normal, and MAO-A deficiency was excluded. III-4 had learning difficulties. III-5 was born at term after an uncomplicated pregnancy with birth weight 4400 grams. Developmental delay was evident from early age. At age 12 extensive investigations were performed. At this time he had a moderate to severe ID and severe behavioral problems, refractory to treatment, including Haldol and valproic acid. An EEG showed a diffuse encephalopathy, without epileptic discharges, a brain CT-scan was normal (data not shown). IQ testing age 16 years showed verbal IQ (VIQ) compatible to age 4.7 years, and performance IQ (PIQ) compatible to 4.1 years. He showed minor regression when he was retested at 28 years with the Wechsler Intelligence Scale (WISC) when his cognitive abilities were compatible to a 3.5 year-old. His social-emotional functioning was lower, at the level of a 6–18-month-old. Currently, at age 43 years, he is physically healthy, height is 181 cm and weight 90 kg. Neurological examination showed no abnormalities. He has good hearing and vision, with only mild hypermetropia (1.25 dpt). He is independent in basic daily activities such as dressing and eating, but needs incentive. He is on risperidone treatment (0.5 mg twice daily) and laxantia. He communicates mostly through pictograms. He is quiet and introverted, with occasional verbal aggression, but not physical. He likes puzzles. He is easily distracted. He is suspected of autism spectrum disorder but has not been formally tested. Laboratory investigations in the past, including metabolic screen, Fragile X and chromosome studies were normal. III-6: During pregnancy her mother had several hospital admissions due to vaginal bleeding and premature contractions. Chromosome studies in chorion villi biopsy were normal (46, XX). She was born at term, with cleft lip/palate, her birth weight was 2785 grams and length 50 cm. She has mild learning difficulties, her OFC is 54 cm. She has three daughters, in whom no molecular testing was performed. She has had two first trimester miscarriages. The eldest daughter (IV:1) was born at 38 weeks of gestation, BW 2900 gram. She has learning difficulties and receives special education, her OFC is 57 cm. She is overweight. Her second daughter (IV:2) was born with ventricular septal defect, her BW was 2700 grams after 38 weeks of gestation. She has normal development, and no learning difficulties. Chromosome studies in chorion villi biopsy in the middle and youngest daughter were normal (46, XX). The youngest is still too young to assess her development. III-8: the pregnancy was complicated with episodes of vaginal bleeding and premature contractions. Developmental delay became evident in the first year of life. He had psychiatric consultation at age 11, due to problematic and aggressive behavior. Two years later he was diagnosed with a pervasive developmental disorder. Currently, at age 36, he has severe ID and autism spectrum disorder. He has a fixation on mirrors and sunshades, and will go around and close them. He has shown severe aggressive outbursts with destructive behavior towards furniture and other objects. He receives risperidone 2.5 mg daily. WISC testing age 20 years showed VIQ compatible to age 4.7 years, and PIQ compatible to 4.1 years. He was last tested at age 34 (Vineland Adaptive Behavior Scale) and he had shown mild regression over a 3-year period. His adaptive skills are now at a level for communication of a 1 year 7 month old, for daily activities at a 3-year-old level, for motor skills at a 1 year 5 month old level, and socialization skills compatible to a 10 month old. He is physically in good health. He underwent adenotomy, strabismus surgery, and excision of a follicular jaw cyst. His current height is 177 cm, and weight 82 kg. Neurological examination showed only brisk reflexes of the lower extremities. He has normal hearing and myopia, with normal media and fundi. Fragile X testing was negative.

**Table 1:** **Summary of clinical features present in affected males and females in the MRX78 family**.

Subject	Gender	Intellectual disability	Epilepsy	Behavioral problems
I-2	F	Mild, illiterate	–	–
II-2	F	Mild	–	–
II-6	F	Present	–	–
II-7	M	Learning difficulties*	–	–
II-8	F	Mild	–	–
II-9	M	Severe, does not speak	Present	Does not interact socially
III-1	M	Severe, does not speak	One seizure as a teenager	Aggressive against others
III-2	M	Severe, does not speak	–	Aggressive against others
III-3	M	Moderate	–	–
III-4	F	Learning difficulties	–	–
III-5	M	Moderate-severe	Diffuse encephalopathy without epileptic discharge	Occasional verbal aggression, suspected of ASD but not formally tested
III-6	F	Learning difficulties	–	–
III-8	M	Severe	–	Pervasive developmental disorder, ASD, severe aggressive outbursts against objects
IV-1	F	Learning difficulties	–	–

*Cause unknown.

### Identification of a p.A789V Mutation in IQSEC2 in Family MRX78

X-chromosome exome sequencing followed by bioinformatics analysis and filtering against publicly available datasets revealed three novel missense changes: *IQSEC2*, chrX:53277996G>A, p.A789V, consensus score 4.06, predicted as probably damaging (PolyPhen-2) and damaging (SIFT) with a CADD score of 19; *TXLNG*, chrX:16859571G>C, p.E423D with a low conservation score (0.02) and predicted as benign (Polyphen-2) and tolerated (SIFT) and CADD of 16; *WAS*, chrX:48542814T>G, donor, conservation score 3.69, and CADD of 12. This strongly suggested that the novel missense mutation identified in *IQSEC2* could be responsible for XLID in this family. Subsequent segregation analysis using Sanger DNA sequencing indicated that the *IQSEC2* chrX:53277996G>A variant co-segregated with the XLID phenotype in all individuals tested (Figures 1A,B).

### IQSEC2 p.A789V Mutation is Predicted to Disrupt SEC7 Domain Folding

**Figure 2:**
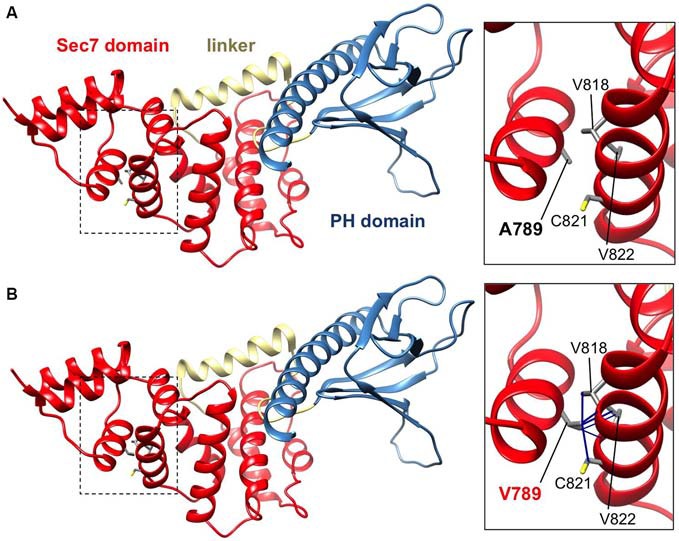
**IQSEC2 A789V mutation is predicted to disrupt SEC7 domain folding.** Side views of the molecular models of IQSEC2 Sec7 and PH domains, with the Sec7 domain in red, the PH domain in blue and the linker between them in gold. Boxed areas show the areas expanded in the inserts to the right. **(A)** Normal IQSEC2 structure, and **(B)** mutant IQSEC2 harboring the p.A789V mutation. Note that the larger valine side-chain in the packed hydrophobic pocket between helices is predicted to cause numerous clashes with surrounding side-chains and/or backbones of residues V818, C821 and V822, indicating a potential disruption of the local fold in this region.

IQSEC2 has a multi-domain structure consisting of a regulatory IQ-like motif, a catalytic Sec7 domain, a pleckstrin homology (PH) domain and a PDZ binding motif (Figure 1C). Residue A789 is located in the catalytic Sec7 domain of IQSEC2 and is highly conserved within IQSEC1–3 (Figure 1D), whereas seven out of nine rare missense variants identified in this domain in normal controls (ExAC database^5^) are mostly present in single individual, and are not conserved residues between IQSEC1−IQSEC3. It is also notable that 3 of 4 previously reported *IQSEC2* missense mutations identified in XLID families are located in the Sec7 domain (Figure 1C; Shoubridge et al., 2010), including the previously investigated R863W mutation present in the large family MRX1. This R863 residue is the only highly conserved amino acid that is substituted to R863Q in a single normal female. To visualize the potential structural consequences of the p.A789V change, we built a comparative model of the IQSEC2 Sec7 and PH domains using the structures of three sequence-related ArfGEFs: BIG1, GEP100 and Grp1 (PDB ID: 3LTL, 3QWM). The resulting model revealed that A789 is located on the third short helix of nine in the Sec7 domain, although it is not one of the residues predicted to interact with Arf GTPases, GDP or Zn^2+^ binding (Figures 1D, 2A). The third and fourth helices come together closely and appear to form a hydrophobic pocket. The residues on these two helices are highly conserved and form a very similar structure in all Sec7-containing crystal structures in PDB, suggesting that the interaction between these helices may be of importance for proper folding of the Sec7 domain. Substitution of alanine at residue 789 with valine introduces a larger side-chain into this packed hydrophobic pocket between helices, which is predicted to cause numerous clashes with surrounding side-chains and/or backbones of residues V818, C821 and V822 (Figure 2B).

### Mutation p.A789V in IQSEC2 Disrupts ArfGEF Activity

**Figure 3:**
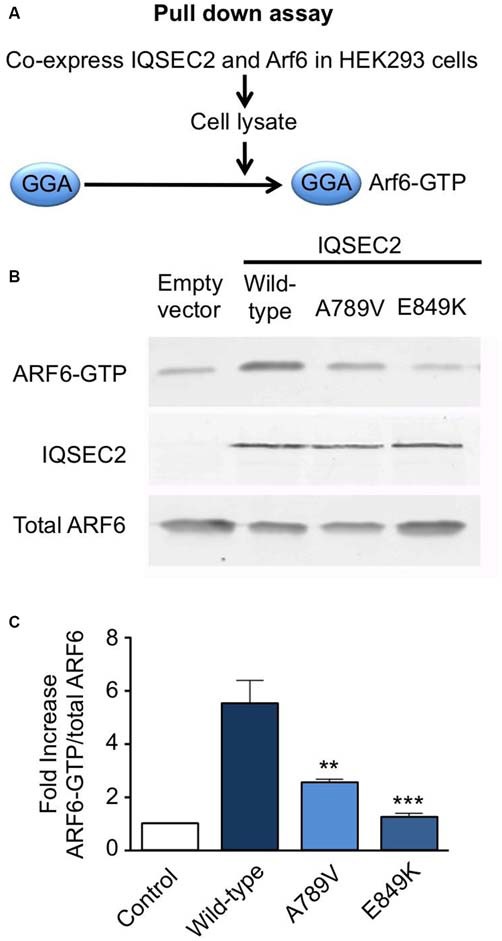
**Mutation A789V in IQSEC2 disrupts ArfGEF activity. (A)** GGA pulldown assay: HA-tagged ARF6 and FLAG-tagged wild-type or IQSEC2^A789V^ or IQSEC2^E849K^ (control) were transfected into HEK293 cells and lysates were subjected to a pull-down assay with GST:golgi-localized, ear-containing arf-binding protein 3 (GGA3) to isolate ARF6-GTP. **(B)** The precipitates (top row) and lysates (bottom row) were probed with anti-HA to detect ARF6 and anti-FLAG to detect expression of IQSEC2 (middle row). **(C)** ARF6-GTP levels were normalized to total ARF6 levels and are depicted as the fold-increase over ARF6 transfected with empty vector. The GGA pulldown assay was assessed by one-way ANOVA (*F* = 15.69, *p* < 0.001) followed by Tukey’s Multiple Comparison Test for *post hoc* analysis (significance indicated in **C**). Error bars in the bottom panel represent the standard error of the mean (SEM) from three independent experiments. **p ≤ 0.01; ***p ≤ 0.001.

We also tested the GEF activity of full-length IQSEC2 and the IQSEC2^A789V^ mutant in a cellular model using a pull-down assay using the adaptor protein Golgi-localized, ear-containing ARF-binding protein 3 (GGA3; Figure 3A). GGAs specifically interact with active, GTP-bound ARF but do not interact with inactive ARF GTPases. As a control, we used the IQSEC2^E849K^ dominant-negative mutation, which reduces the exchange activity of the IQSEC2 Sec7 domain by several orders of magnitude (Shoubridge et al., 2010). Co-transfection of ARF6 with wild-type IQSEC2 resulted in a ~5-fold increase (5.5 ± 0.91, mean ± SEM, *n* = 3) in GTP-bound ARF6, but only a 2.5- to 1-fold increase when expressed with IQSEC2^A789V^ (2.5 ± 0.19) or IQSEC2^E849K^ (1.2 ± 0.19) respectively (Figures 3B,C). This is consistent with a significant loss of ArfGEF activity in the IQSEC2^A789V^ mutant. It is also noteworthy that the IQSEC2^A789V^ mutation does not appear to affect protein stability (Figure 3B), at least in cellular models.

## Discussion

This study reports the identification and functional characterization of a novel mutation (c.C2366T, p.A789V) in *IQSEC2* that based on variant filtering and segregation analysis likely presents the cause of XLID in the large family MRX78. A previous analysis in this family revealed X-linked inheritance with linkage to a 15.16 cM region on Xp11 (de Vries et al., 2002). Using molecular modeling of wild-type and mutant protein and assays for ArfGEF activity, we were able to establish the likely pathomechanism for p.A789V: a disrupted hydrophobic pocket in the Sec7 domain structure, leading to loss of IQSEC2 ArfGEF activity. The identification of *IQSEC2* as the causative gene for MRX78 is consistent with previous studies that have identified IQSEC2 as a neuronally-expressed ArfGEF with a key role in excitatory synaptic transmission. At excitatory synapses, IQSEC2 interacts with multivalent postsynaptic density (PSD) proteins such as IRSp53 (Sanda et al., 2009) and PSD-95 (Sakagami et al., 2008), forming a complex with *N*-methyl-D-aspartate (NMDA) receptors.

A recent reassessment of the MRX78 family by one of the co-authors (RO) revealed that except for one all affected males presented with moderate to severe ID, several affected males did not acquire speech skills and presented with behavioral disturbances. Females from this family who carry the mutation on one of her X-chromosomes presented with learning difficulties or mild ID. A summary with genetic findings and clinical presentations of other affected individuals who carry missense, nonsense, deletions, insertions and complex rearrangements is given in (Table 2). Shoubridge et al. (2010) initially reported four missense mutations in *IQSEC2* associated with non-syndromic XLID (IQ domain p.R359C, Sec7 domain p.R758Q, p.Q801P and p.R863W). However, seizures, autistic traits, psychiatric problems and delayed early language skills were noted in different male individuals in this study. Furthermore, several *de novo* deleterious *IQSEC2* mutations have been reported in males and females who presented with seizure disorders, including Rett-like syndrome and Lennox-Gastaut syndrome: a frameshift mutation (p.N91Kfs*112) and a truncating chromosome breakpoint in intron 1 [46, X(tX;20)(p11.2;q11.2)] in the N-terminal part of IQSEC2 (Morleo et al., 2008; Olson et al., 2015), a two base pair deletion resulting in p.C684X (region between IQ-like motif and Sec7 domain) (Gandomi et al., 2014), two additional mutations affecting the Sec7 domain (p.R855X and p.S861T that is predicted to abolish the native splice donor site; Rauch et al., 2012; Gandomi et al., 2014; Tran Mau-Them et al., 2014), and one *de novo* mutation (p.Q1108X) C-terminal to the PH domain (Allen et al., 2013). Additionally, Tran Mau-Them et al. (2014) reported two severely affected males with Rett-like features who carried *de novo* intragenic duplications, both of which were thought to disrupt *IQSEC2*. Furthermore, a p.I888Afs followed by a subsequent premature stop codon has been identified in a male with severe non-syndromic ID (Tzschach et al., 2015). IQSEC2 mutations in ID patients which affect the PH domain of the protein have also been reported, including a p.L992X change in a patient with schizophrenia (Purcell et al., 2014), a *de novo* p.Q1033X mutation in a male with severe ID, epilepsy, strabismus and autistic features (Redin et al., 2014), and a *de novo* mutation (p.R1055X) in a female with severe ID, epilepsy and borderline macrocephaly (Tzschach et al., 2015). A partial *de novo* duplication of TENM3 inserted into *IQSEC2* and subsequent formation of an in-frame IQSEC2-TENM3 fusion gene was also recently reported. The resulting disruption of *IQSEC2* was thought to contribute to ID, epilepsy, progressive spasticity, and microcephaly in that patient (Gilissen et al., 2014). Furthermore, three maternally inherited duplications with disrupted *IQSEC2* in five males who presented with ID and behavioral disturbances were reported by Moey et al. (2015), and a *de novo* deletion of *IQSEC2* and the XLID gene *KDM5C* (MIM*314690; Jensen et al., 2005) has been identified in a girl with severe ID and autistic behavior (Fieremans et al., 2015). Although originally described as non-syndromic XLID, newly acquired clinical data from the MRX78 family suggests that additional features might be associated with the IQSEC2 p.A789V mutation, including variable seizures in males, which is consistent with other reports, and behavioral disturbances in five out of six affected males. By contrast, heterozygous female have learning disabilities. As *IQSEC2* is one of the few genes that escape X-inactivation in females with an expression level similar in males and females (Moey et al., 2015) we hypothesize that, in these female carriers, the missense mutation is sufficient to produce symptoms but that the mutant protein has residual function and in addition that there is some compensation from the normal allele. This is in contrast to early truncating mutations in *IQSEC2*, which in females are associated with a more severe phenotype including infantile spasms, epilepsy, autistic features, Lennox Gastaut syndrome and Rett-like syndrome. Given that in several severely affected females *IQSEC2* is either deleted on one of the X-chromosomes or the truncating change is located early in the N-terminus and therefore can be assumed to result in protein degradation due to nonsense-mediated mRNA decay, it is unlikely that in these cases the mutations produce a dominant negative effect. Thus, it is very likely that *IQSEC2* is a dosage-sensitive gene that needs to be tightly regulated for normal cognitive function in males and females and that *IQSEC2* mutations can cause a spectrum of clinical features in both sexes.

**Table 2:** ***IQSEC2* mutations and associated phenotypes**.

Missense and nonsense mutations				
Nucleotide	Protein	Gender of affected and inheritance	Reported phenotype	Reference
c.1075C>T	p.R359C	4M and 1F, maternally inherited	Mild to moderate ID in males, non-syndromic^†^, female with learning difficulties	Shoubridge et al. (2010)
c.2273G>A	p.R758Q	8M, maternally inherited	Mild ID, non-syndromic^†^	Shoubridge et al. (2010)
c.C2366T	p.A789V	6M and 7F (MRX78), maternally inherited	Moderate to severe ID and behavioral disturbances in males, mild ID and learning difficulties in females	This study
c.2402A>C	p.Q801P	8M and 2F (MRX18), maternally inherited	Moderate to severe ID in males, non-syndromic^†^, carrier females with learning difficulties	Gedeon et al. (1994) and Shoubridge et al. (2010)
c.2563C>T	p.R855X	1M, *de novo*	ID, epilepsy, stereotypic hand movements, strabismus, language partially acquired but delayed, both language and motor skills regressed, behavioral disturbances including self-injury, abnormal MRI	Rauch et al. (2012) and Tran Mau-Them et al. (2014)
c.2582G>C	p.S861T, predicted to abolish splice site	1M, *de novo*	ID, developmental delay, seizures, hypotonia, vision impairments, plagiocephaly, autistic-like features, absent language skills, abnormal MRI	Suthers et al. (1998) and Gandomi et al. (2014)
c.2587C>T	p.R863W	12M (MRX1), maternally inherited	Moderate ID, non-syndromic^†^	Turner et al. (1971), Suthers et al. (1998), and Shoubridge et al. (2010)
c.2975T>A	p.L992X	1, gender not reported	Schizophrenia	Purcell et al. (2014)
c.3097C>T	p.Q1033X	1M, *de novo*	Severe ID, no speech, motor developmental delay, severe epilepsy, strabismus, autistic features	Redin et al. (2014)
c.3322C>T	p.Q1108X	1F, *de novo*	Lennox-Gastaut syndrome, global developmental delay	Allen et al. (2013)
c.3163C>T	p.R1055X	1F, *de novo*	Severe ID, epilepsy, borderline macrocephaly	Tzschach et al. (2015)
**Small deletions/insertions**				
**Nucleotide**	**Protein**	**Gender and Inheritance**	**Reported phenotype**	**Reference**
c.273_282del	p.N91KfsX112	1F, *de novo*	ID, features of Rett syndrome, no epilepsy, gait abnormalities, stereotypic hand movements, regression but did not lose purposeful hand skills, cranial MRI showed delayed myelination.	Olson et al. (2015)
c.2052_2053delCG	p.C684X	1M, *de novo*	ID, hypotonia, strabismus, astigmatism, cortical vision impairment, hypoplastic corpus callosum, myoclonic seizures, positional plagiocephaly with mild relative microcephaly, stereotypic hand movements	Gandomi et al. (2014)
c.2662dup	p.I888AfsX16	1M, *de novo*	Severe ID, non-syndromic	Tzschach et al. (2015)
**Gross deletions and insertions without *HUWE1* gene involvement**				
**Nucleotide**		**Gender and Inheritance**	**Reported phenotype**	**Reference**
Deletion 400 kb also including *KDM5C*		1F, *de novo*	Severe ID and autistic-behavior	Fieremans et al. (2015)
Duplication 22 kb incl. ex. 3		1M, *de novo*	Severe ID, language partially acquired, midline stereotypic hand movements, partial epilepsy, regression of language and motor skills, behavioral disturbances	Tran Mau-Them et al. (2014)
Duplication 42 kb incl. ex. 3–7		1M, *de novo*	Severe ID, postnatal microcephaly, no speech, no purposeful hand skills, seizures, behavioral disturbances	Tran Mau-Them et al. (2014)
62 kb insertion of *TENM3* sequence, fusion gene		1F?, *de novo*	Severe ID, microcephaly, epilepsy, progressive spasticity, small hands and feet, poor vision	Gilissen et al. (2014)
Duplication 361 kb with disrupted *IQSEC2* long isoform		1M, maternally inherited	Language delay and behavioral problems	Moey et al. (2015)
Duplication 403 kb with short IQSEC2 isoform duplicated		3M, maternally inherited	Mild-moderate ID, mild learning difficulties in one male, autism spectrum disorder, obsessive behavior	Moey et al. (2015)
Duplication 579 kb, including *TSPYL*, *KDM5C* and *IQSEC2*		1M, maternally inherited	Global delay, severe expressive speech delay, behavioral disturbances	Moey et al. (2015)
**Complex rearrangements**				
**Description**			**Reported phenotype**	**Reference**
Balanced 46, X, t(X;20) (p11.2;q11.2) *de novo*			Severe ID, delayed language and motor development, infantile spasms, regression	Morleo et al. (2008)

*^†^seizures, autistic traits, psychiatric problems and delayed early language skills were noted in different individuals in this study, although none of these additional phenotypes were consistent in all affected male individuals in the families studied*.

## Author Contributions

VMK, RW and RJH designed the experiments; AJMH and RO contributed DNA samples and clinical evaluation; VMJ, MLH, PL, CJ, MB, MT and KH performed the experiments; VMK, HH, SAH, MT, KH, RW, and RJH analyzed the data; VMK, RW and RJH wrote the paper. All authors were involved in revising the paper for important intellectual content, and gave final approval of the version to be published.

## Funding

This work was supported by the Medical Research Council (J004049 to RJH and KH) and the EU FP7 project GENCODYS, grant number 241995 (to HH and VMK) and the Whitehall Foundation (grant number 2010-08-68 to RSW). The funders had no role in study design, data collection and analysis, decision to publish, or preparation of the manuscript. The authors would like to thank the Exome Aggregation Consortium and the groups that provided exome variant data for comparison. A full list of contributing groups can be found at http://exac.broadinstitute.org/about.

## Conflict of Interest Statement

The authors declare that the research was conducted in the absence of any commercial or financial relationships that could be construed as a potential conflict of interest.

## Footnotes

^1^http://toolkit.tuebingen.mpg.de/hhpred

^2^http://zhanglab.ccmb.med.umich.edu/I-TASSER

^3^http://bioinf.cs.ucl.ac.uk/psipred/

^4^http://raptorx.uchicago.edu/

^5^http://exac.broadinstitute.org/gene/ENSG00000124313
